# 

**Published:** 1820

**Authors:** 


### GENERAL INDEX

TO THE

### LONDON MEDICAL AND PHYSICAL JOURNAL,

FROM VOLUME ONE TO FORTY, INCLUSIVE;

COMPRISING

**An Analytical Table of their Contents,**

*ARRANGED IN ALPHABETICAL ORDER,*

WITH

REFERENCES TO THE WHOLE OF THE CITED AUTHORITIES, UNDER THEIR NOMINAL CHARACTERS, &c.

Surgeon Genl's Office
5147
LIBRARY.
Washington D.C.

*LONDON:*

PRINTED FOR JOHN SOUTER, 73, ST. PAUL'S CHURCH-YARD;

By J. and C. Adlard, 23, Bartholomew Close.

1820.

W1
LO31

A

### SUPPLEMENT

TO THE

### FIRST GENERAL INDEX

TO THE

### LONDON MEDICAL AND PHYSICAL JOURNAL;

COMPRISING

**An Analytical Table of the Contents**

OF THE

VOLUMES FROM XXXV. TO XL. INCLUSIVE.

